# Assessing the Use of GACOS Products for SBAS-InSAR Deformation Monitoring: A Case in Southern California

**DOI:** 10.3390/s19183894

**Published:** 2019-09-10

**Authors:** Qijie Wang, Wenyan Yu, Bing Xu, Guoguang Wei

**Affiliations:** School of Geosciences and Info-Physics, Central South University, Changsha 410083, China; qjwang@csu.edu.cn (Q.W.); ywy185011006@csu.edu.cn (W.Y.); 175011007@csu.edu.cn (G.W.)

**Keywords:** InSAR, Short Baseline Subset (SBAS), deformation monitoring, GACOS products

## Abstract

The Generic Atmospheric Correction Online Service (GACOS) products for interferometric synthetic aperture radar (InSAR) are widely used near-real-time and global-coverage atmospheric delay products which provide a new approach for the atmospheric correction of repeat-pass InSAR. However, it has not been determined whether these products can improve the accuracy of InSAR deformation monitoring. In this paper, GACOS products were used to correct atmospheric errors in short baseline subset (SBAS)-InSAR. Southern California in the U.S. was selected as the research area, and the effect of GACOS-based SBAS-InSAR was analyzed by comparing with classical SBAS-InSAR results and external global positioning system (GPS) data. The results showed that the accuracy of deformation monitoring was improved in the whole study area after GACOS correction, and the mean square error decreased from 0.34 cm/a to 0.31 cm/a. The improvement of the mid-altitude (15–140 m) point was the most obvious after GACOS correction, and the accuracy was increased by about 23%. The accuracy for low- and high-altitude areas was roughly equal and there was no significant improvement. Additionally, GACOS correction may increase the error for some points, which may be related to the low accuracy of GACOS turbulence data.

## 1. Introduction

Interferometric synthetic aperture radar (InSAR) is a powerful technique for topographic and ground surface deformation mapping [1,2] which enables all-weather, non-contact, wide spatial coverage with centimeter- or even millimeter-scale monitoring of the study area [3,4,5,6]. InSAR has been widely used for fine-resolution mapping and other remote sensing applications over the past two decades [7,8,9]. However, the application of InSAR is limited by atmospheric delay, orbit errors, topographical errors, and so on [10,11]. One of the most intractable limitations is the effect of the atmosphere on repeat-pass InSAR. Researchers have devoted many efforts to removing the effect of atmospheric delay [12,13,14], but the current methods are still vulnerable to poor spatial and temporal resolution and accuracy [15]. 

Auxiliary atmospheric datasets or models, such as global positioning system (GPS) atmospheric measurements [12,16], moderate-resolution imaging spectroradiometer (MODIS) and medium-resolution imaging spectrometer (MERIS) techniques [17,18], and numerical meteorological models [19] are commonly used to estimate atmospheric delay. However, GPS stations are sparsely distributed, spectroradiometers are only applicable in cloud-free daylight conditions, and the meteorological models have high potential uncertainties. In recent years, Zhenhong Li and colleagues released the Generic Atmospheric Correction Online Service (GACOS) atmospheric delay products, which are based on a fusion of GPS measurements and weather models [20,21,22]. With their global coverage and high temporal and spatial resolutions, GACOS products provide a new path for atmospheric correction of repeat-pass InSAR. However, whether GACOS atmospheric products can improve the accuracy of deformation monitoring in time-series InSAR needs to be further validated. 

The purpose of time-series InSAR techniques is to estimate geophysical parameters after the error sources are reduced by analyzing the time series of SAR images or interferograms [23]. The two most representative methods for analyzing time-series InSAR data are permanent scattering InSAR (PS) [24,25] and small baseline subset (SBAS) [26]. In general, PS-InSAR only focuses on pointlike coherent targets, which exhibit highly stable backscattering behavior and usually correspond to man-made structures, artificial reflectors, or bare rocks. In contrast, SBAS-InSAR employs distributed targets, which contain more random scatters and can be found in rural environments. Further, SBAS-InSAR effectively reduces decoherence effects with short spatial–temporal baselines. Therefore, SBAS-InSAR technology is more practical and reliable. Since the development of these methods, many scholars have continuously applied SBAS-InSAR technology to monitor urban surface subsidence, volcanic movement, glacier motions, and landsides [27,28,29,30]. Although SBAS-InSAR technology has made remarkable progress in improving the accuracy of velocity estimates, the effect of the troposphere still needs to be resolved [31]. In this paper, SBAS-InSAR was taken as an example to analyze the corrective effect of GACOS products on time-series InSAR. The atmospheric delay that impacted the SBAS-InSAR was estimated using the GACOS products (GACOS-based SBAS-InSAR for short), and we compared the results of GACOS-based SBAS-InSAR with those of classical SBAS-InSAR and GPS projected in the radar line of sight (LOS) direction to determine the effectiveness of GACOS products in correcting atmospheric delay in time-series InSAR.

This paper is organized as follows. Section 2 provides a brief introduction of the GACOS products and the method of GACOS-based SBAS-InSAR. Taking southern California as an example, Section 3 introduces the experimental data and processing flow. In Section 4, GACOS-based SBAS-InSAR results, classical SBAS-InSAR results, and GPS data are compared, and the results are analyzed and illustrated. Finally, Section 5 provides our conclusions, summarizing the main findings of this study.

## 2. Methods

### 2.1. The GACOS Products

Tropospheric delay, which can be expressed as the spatial–temporal delay uncertainty, is often considered to be the sum of a stratified component highly related to topography and a turbulent component resulting from interference processes (e.g., severe weather) [11,32,33]. The GACOS atmospheric products, for the correction of InSAR and other measurements, use the iterative tropospheric decomposition (ITD) interpolation model to separate the elevation-related signals and turbulence signals from the zenith total delay (ZTD) and interpolate this to generate high-resolution tropospheric delay maps. The ITD model is defined as [21]

(1) $$ZTD_{k}=T\left(x_{k}\right)+L_{0}e^{-\beta {\bar{h}}_{k}}+\varepsilon_{k}$$

The ZTD of point k consists of the turbulence signal, the elevation signal, and the residual error. *T* represents the turbulence signal, which is composed of medium- and long-wavelength signals interpolated by the inverse distance weighting (IDW) method, and $x_{k}$ denotes the station coordinate vector in the local geocentric coordinate system. The exponential function with coefficient β is related to the elevation-correlated signal; $L_{0}$ is the elevation-correlated delay at sea level for the selected area; $\bar{h_{k}}$ represents the scaled height, which is calculated as $\overset{-}{h_{k}}=(h_{k}-h_{\min})/(h_{\max}-h_{\min})$; and $\varepsilon_{k}$ denotes the unmodeled residual error. The assumption of the ITD model is that the elevation-related component obeys the exponential law and the turbulent component obeys the IDW interpolation law. However, the elevation-related component and turbulence component are not tightly integrated, and they account for very different proportions of the ZTD. The ITD model has been implemented in GACOS, which automatically generates correction maps for user requests. In GACOS products, shuttle radar topography mission (SRTM) digital elevation model (DEM) data are used in the range from 60° south latitude to 60° north latitude (S60–N60), and Advanced Spaceborne Thermal Emission and Reflection Radiometer (ASTER) global digital elevation model (GDEM) data are used in N60–N83 and S60–S83.The high-resolution European Centre for Medium-Range Weather Forecasts (ECMWF) weather model with 0.125° and 6 h resolutions was adopted in GACOS.

Yu et al. showed that ~1 cm ZTD quality can be obtained in the real-time mode. Eight global distribution interferograms (250 × 250 km) were used to evaluate the ITD models [22]. The average improvements in the phase standard deviation (StdDev) obtained from the atmospheric correction maps were 47%, 49%, and 54% for GPS, ECMWF, and integrated corrections, respectively. The corrected InSAR deformations in the LOS direction were also compared with GPS displacement. The RMS values of the GPS, ECMWF, and integrated corrections were improved by 55%, 45%, and 63%, respectively. Therefore, the performance of the integrated model was the best. The combination of different data sources improved the reliability of the model. Considering the displacement StdDev and RMS difference produced by the correction interferogram, the difference was about 1 cm.

GACOS has the following key features: globally available, operational in a near-real-time mode, easy to implement, and users are informed how the model performs and whether the correction is recommended. GACOS products are given in a grid binary format, and a ReadMe file is provided to demonstrate how to use GACOS tropospheric correction maps.

GACOS products are based on a combination of GPS and weather model data and provide high-spatial-resolution zenith total delay maps to be used for correcting InSAR measurements and other applications. In the following sections, GACOS products were assessed in practical application to time-series InSAR.

### 2.2. InSAR Atmospheric Correction Based on GACOS Products

Atmospheric correction based on GACOS products is achieved by calculating the difference between the InSAR interferogram and the GACOS atmospheric product after processing, which is used to weaken or eliminate the influence of atmospheric error in the interferogram. 

The interferogram obtains the deformation in the LOS direction, while GACOS products correspond to the atmospheric delay in the zenith direction. The GACOS products need to be converted to the phase delay in the LOS direction. The conversion formula is as follows:

(2) ϕGACOS=4πλ⋅GACOScut/cosθinc

where $GACOS_{cut}$ is the clipped GACOS atmospheric product data, $\theta_{inc}$ is the radar incidence angle, and λ is the radar central wavelength.

Alongside this, GACOS products should be geocoded in the SAR coordinate system (range-Doppler SAR processing) to obtain the atmospheric delay maps of the interferograms. If the SAR images at times $t_{i}$ and $t_{j}$ constitute the interferometric pairs, and we assume that $t_{j}>t_{i}$, the atmospheric correction phase of the interferogram at pixel (x,y) can be expressed as

(3) $$\delta f_{GACOS,i,j}\left(x,y\right)=f_{GACOS,j}(x,y)-f_{GACOS,i}(x,y)$$

The interferogram based on GACOS atmospheric correction is obtained by the difference between the unwrapped interferogram and the corresponding atmospheric correction map.

### 2.3. The GACOS-Based SBAS-InSAR Method

The basic principle of GACOS-based SBAS-InSAR is to take the single InSAR deformation results corrected by the GACOS atmospheric product as the observed values and process these data using the SBAS technique.

Firstly, N+1 SAR images in the same region are projected into the same main image coordinate system, and then *M* multi-looked interferometric pairs are generated based on the condition that the spatial–temporal baseline is lower than a certain threshold [26]. *M* represents the number of interferograms which satisfy the requirement

(4) $$\frac{N+1}{2}\leq M\leq N(\frac{N+1}{2})$$

The interference phase of the interferogram at pixel (*x*, *y*) can be expressed as [23]

(5) $$\delta \phi_{i,j}\left(x,y\right)=\frac{4\pi }{\lambda }d_{i,j}+\frac{4\pi }{\lambda }\frac{B_{⊥i,j}}{R_{i}\sin\theta \left(x,y\right)}\Delta h(x,y)+\phi_{atm,i,j}\left(x,y\right)+\phi_{orbit,i,j}\left(x,y\right)+\phi_{n,i,j}\left(x,y\right)$$

where λ is the central wavelength of the radar; $d_{i,j}$ is the cumulative deformation of the LOS direction relative to the reference time; $B_{⊥i,j}$ is the perpendicular baseline of the two images; R is the distance from the sensor to the measurement area; x and y are the coordinate values of the pixels in the distance and azimuth directions; Δh(x,y) is the residual terrain phase at pixel (x,y); and $\phi_{atm,i,j}(x,y)$, $\phi_{orbit,i,j}\left(x,y\right)$, and $\phi_{n,i,j}(x,y)$ are the atmospheric error, orbit error, and noise distortion at pixel (*x*, *y*), respectively.

In the process of SBAS-InSAR, it is assumed that the random errors have been eliminated by filtering, the orbit errors have been fitted by the quadratic polynomial model and iterative least squares method [34], and the atmospheric delay errors have been eliminated by GACOS atmospheric correction. 

Equation (5) can be simplified as follows:

(6) $$\delta \phi_{i,j}\left(x,y\right)=-\frac{4\pi }{\lambda }d_{i,j}-\frac{4\pi }{\lambda }\frac{B_{⊥i,j}}{R_{i}\sin\theta \left(x,y\right)}\Delta h(x,y)$$

**Figure 1:**
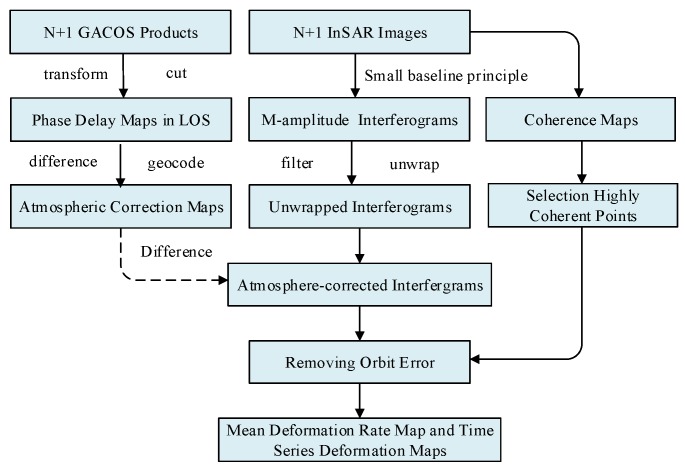
Flow chart of Generic Atmospheric Correction Online Service (GACOS)-based short baseline subset (SBAS)-interferometric synthetic aperture radar (InSAR). LOS: line of sight.

The single InSAR deformation results corrected by the GACOS atmospheric products are then taken as observation values. Based on the minimum norm rule and the singular value decomposition (SVD) decomposition method, deformation time series and average deformation rate maps are obtained. The flow chart of SBAS-InSAR based on GACOS is shown in Figure 1.

## 3. Dataset and Processing

### 3.1. Data Sources

Single-look complex (SLC) images of 27 ENVISAT advanced synthetic aperture radar (ASAR) descending orbit sites in Southern California were selected as the experimental data. The time span of the images was from May 14, 2005 to September 25, 2009. The basic parameters of the ASAR data set are shown in Table 1. In addition, SRTM DEM data with a resolution of 1 arc second were used to remove the terrain phase in the interferogram.

**Figure 2:**
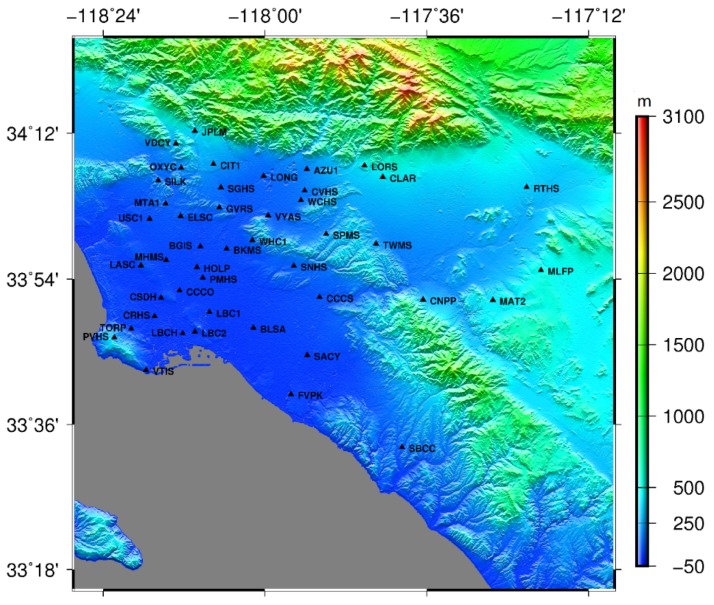
GPS site distribution and digital elevation model (DEM) map of southern California.

The GPS data were downloaded from the Southern California Integrated GPS Network (SCIGN, http://www.scign.org). The SCIGN has built more than 250 GPS continuous observation stations to monitor real-time three-dimensional surface deformation in southern California. 45 GPS continuous observation stations with a uniform distribution in the study area were selected for this experiment (Table 2). And the distribution of the GPS stations is shown in Figure 2. Since InSAR can only measure one-dimensional deformation in the LOS direction, in order to allow a comparison, the three-dimensional deformation measured by GPS was projected into the LOS direction. The projection formula used is as follows:

(7) $$\Delta L=\left[\begin{array}{ccc} \sin(\theta_{inc})\sin(\alpha ) & -\sin(\theta_{inc})\cos(\alpha ) & \cos(\theta_{inc}) \end{array}\right]\left[\begin{array}{c} \delta_{N}\\ \delta_{E}\\ \delta_{U} \end{array}\right]$$

where ΔL represents the surface deformation in the LOS direction between two radar images; α is the azimuth angle of the satellite heading; $\theta_{inc}$ is the radar incidence angle; and $\delta_{N},\delta_{E},\delta_{U}$ represent the displacement of GPS stations in the north, east, and vertical directions, respectively.

**Figure 3:**
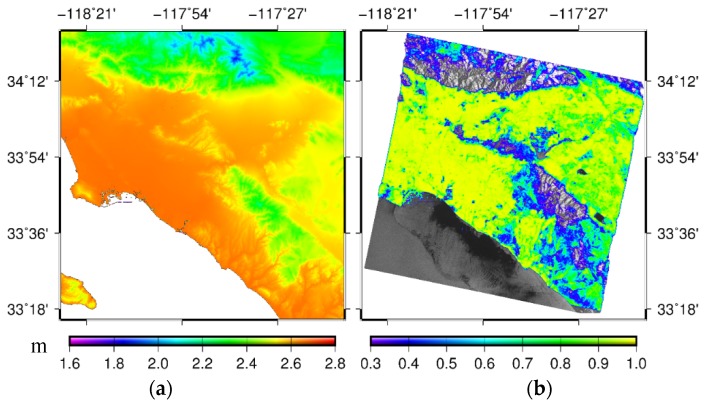
(**a**) GACOS product map at UTC 18:02:00, 14 May 2005 and (**b**) the mean coherence map in southern California of the United States.

To match the InSAR data, 27 GACOS atmospheric delay images with near-real-time SAR images were downloaded from the website (http://ceg-research.ncl.ac.uk/v2/gacos/). Figure 3a shows the GACOS product map at UTC 18:02 on May 14, 2005. It can be seen from Figure 2 and Figure 3a that the delay in GACOS products had an obvious correlation with the elevation stratification, and the atmospheric impact was greater in the lower elevation region than in the higher elevation region. The mean coherence map in southern California of the United States is shown in Figure 3b.

### 3.2. Data Processing

**Figure 4:**
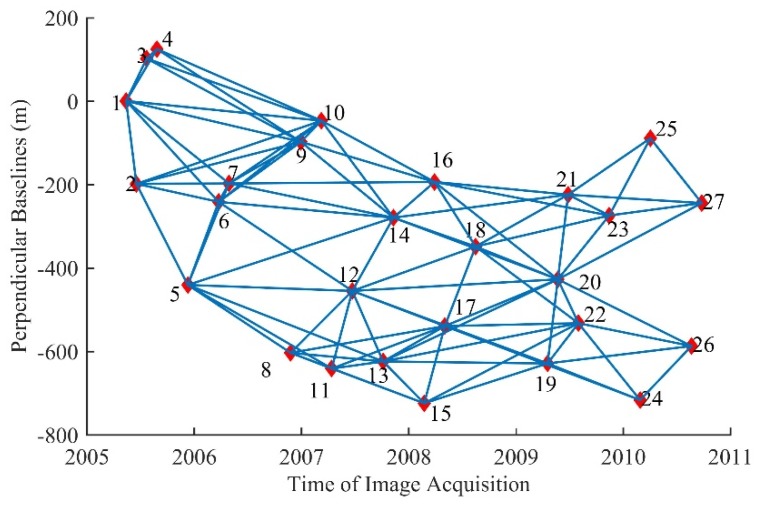
The spatiotemporal baseline map in southern California of the United States.

(1) InSAR data processing. The image of November 10, 2007 was selected as the main image, and other images were registered in the main image coordinate system. A total of 96 interference pairs were obtained with perpendicular baselines shorter than 200 m and temporal baselines shorter than 300 days. The spatial–temporal baseline distribution is shown in Figure 4. The interferometric SAR processor (ISP) module of GAMMA software was used to process the selected interference pairs, and SRTM data were used as an external DEM to eliminate the terrain phase. In order to suppress noise, multilook operations of 2 pixels in range and 10 pixels in the azimuth direction were conducted. Furthermore, an adaptive interferogram filtering algorithm was applied to the multilook interferogram with a 32 × 32 patch size window and 0.5 filter parameter [35]. After that, the minimum cost flow (MCF) algorithm with Delaunay triangulation network was used for phase unwrapping.

(2) Correction of InSAR with GACOS atmospheric correction. In total, 96 atmospheric correction maps were generated according to the steps described in Section 2.2. The interferograms corrected by the GACOS atmospheric correction were obtained by further differencing between the unwrapped interferograms and the atmospheric correction maps. A quadric surface model and iterative least squares method were used to remove residual orbit errors [30]. The sampling distance used in this paper was 20 × 20, and reliable results were usually obtained through two iterations.

(3) Selection of the high-coherence points and solving of the deformation rate. The points with coherence values greater than 0.3, for which the average was greater than 0.5, were selected as the coherent target points. The mean coherence of the study area is shown in Figure 3b. At the selected high-coherence points, GACOS-based SBAS-InSAR was used to establish the observation equation and obtain the results of the surface deformation sequence in the LOS direction.

## 4. Results and Discussion

### 4.1. Comparison of the Deformation Rate Maps

**Figure 5:**
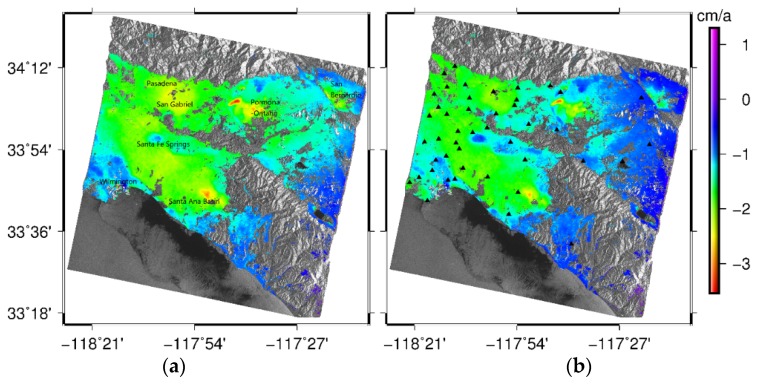
InSAR mean LOS deformation velocity map for southern California for the period from 14 May 2005–25 September 2010 (unit: cm/a). The base image is a grayscale SAR image. (**a**) The classical SBAS-InSAR result. (**b**) The GACOS-based SBAS-InSAR result.

According to the steps described in Section 3.2, the mean deformation rate maps were calculated from May 2005 to September 2010 by classical SBAS-InSAR and GACOS-based SBAS-InSAR, respectively, as shown in Figure 5. 

It can be seen from Figure 5 that the results of the deformation rate obtained by the two methods were highly similar, indicating that the overall surface deformation presented the same trend of subsidence, and the maximum subsidence rate was about −4 cm/a. The surface deformation area caused by human activities (such as groundwater and oil extraction and recharge) was more obvious and relatively consistent, and the seven obvious deformation regions had good consistency with existing research results [28,29]. Although the mean deformation rate obtained by GACOS-based SBAS-InSAR was relatively small compared with that in the classical SBAS-InSAR results in several regions, it could also be clearly distinguished that Santa Ana Basin, Pormona-Ontario, San Bernardio, Pasadena, and San Gabriel have larger sedimentation rates, while the deformation rates of Santa Fe Springs and Wilmington are relatively smaller.

### 4.2. Assessing the GACOS Products Using GPS Data

In order to verify the corrective effect of GACOS atmospheric products in time-series InSAR and to further analyze whether altitude had an impact on the corrective effect, we compared GACOS-based SBAS-InSAR, classical SBAS-InSAR, and GPS projected in the LOS direction, and have briefly evaluated the corrective effect of GACOS atmospheric products and the detection accuracy of the GACOS-based SBAS-InSAR method.

**Figure 6:**
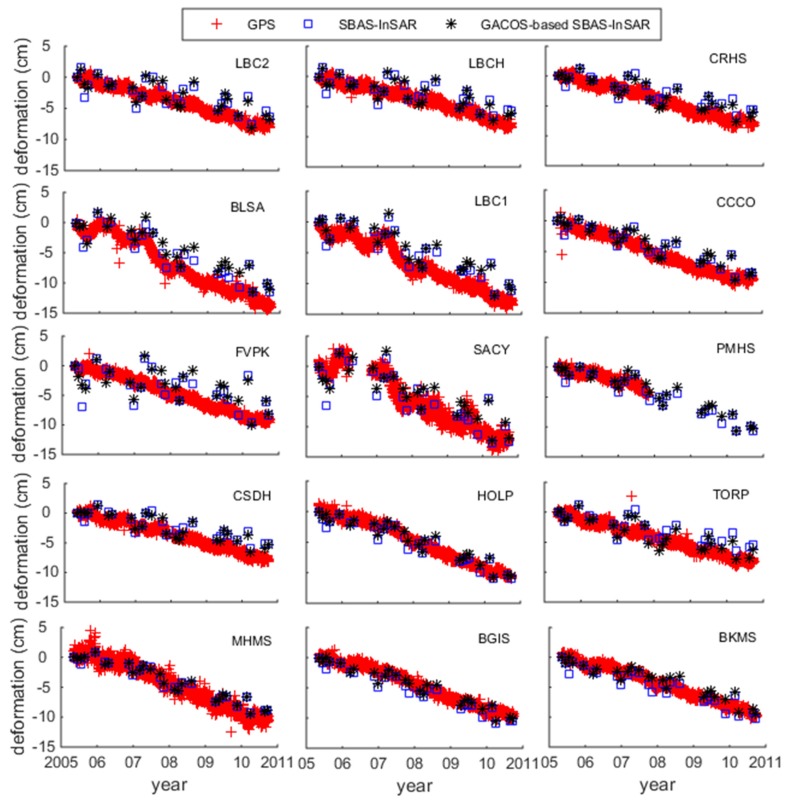
Low-elevation points comparison chart.

**Figure 7:**
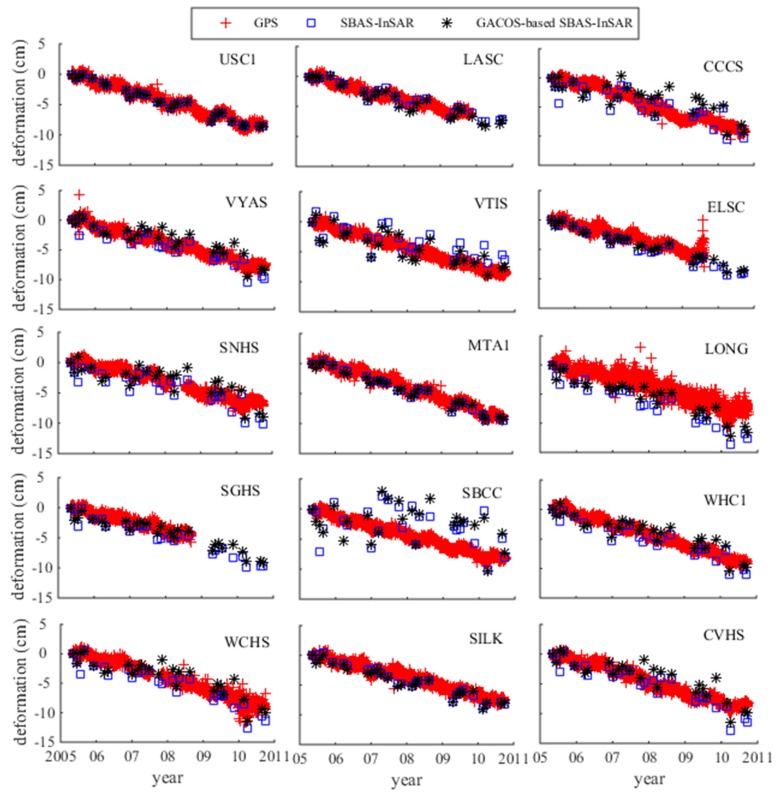
Medium-elevation points comparison chart.

**Figure 8:**
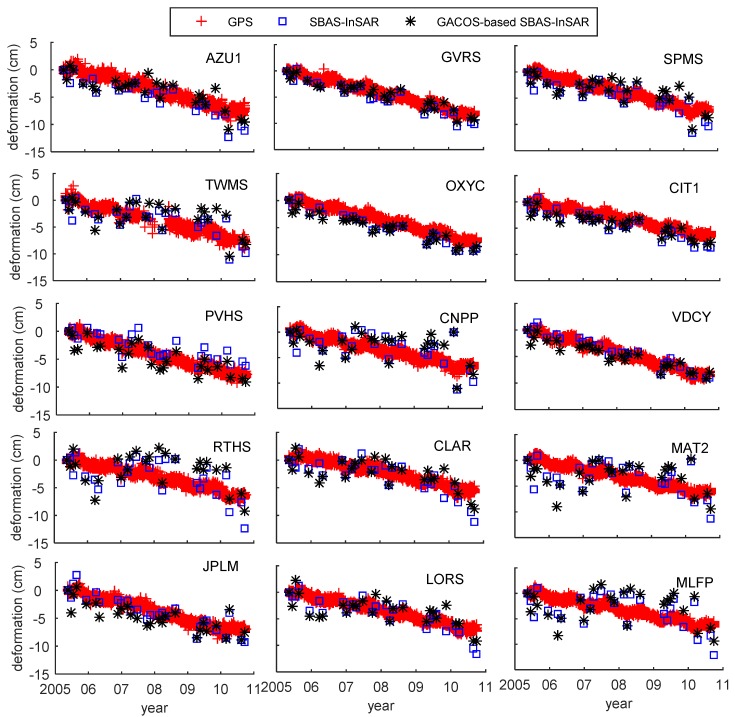
High-elevation points comparison chart.

From Figure 5, it can be seen that the corrective effect of GACOS atmospheric products varied from region to region. In order to further analyze the relationship between the correction effect and the elevation, we divided the GPS point elevation into three parts for comparative analysis (Table 3). We considered the area with elevation less than 15 m as the low-elevation area, which was gentle and coastal. The area with elevation ranging from 15 to 140 m was considered the medium-elevation area, and this area was relatively gentle and far from the coastline. The elevation of the high-altitude area was greater than 140m, and it had large ups and downs and was mostly far from the sea. The deformation sequences are compared in Figure 6, Figure 7 and Figure 8, and the mean deformation rates at high, medium, and low points are listed in Table 4, Table 5 and Table 6, respectively. It was also approximated that the point deformation measured by GPS was equal to the average deformation in the resolution unit measured by InSAR, and the nearest point of a GPS station was selected for comparison.

The time-series deformations of 15 points in the low-altitude region, which were obtained using SBAS-InSAR and GACOS-based SBAS-InSAR, are compared with GPS in the LOS direction in Figure 6. The deformation shown by SBAS-InSAR and GACOS-based SBAS-InSAR in Figure 6 was relatively consistent with that given by GPS points, although the fitting degree of the GPS stations LBC1 and BLSA points after GACOS correction decreased and the GPS station TORP points were more consistent with GPS deformation. It can be seen from the quantitative analysis in Table 4 that the difference between SBAS-InSAR and GPS was −0.53 cm/a to 0.41 cm/a for low-altitude points, and the mean square error was 0.40 cm/a. The difference between GACOS-based SBAS-InSAR and GPS was −0.61 cm/a to 0.29 cm/a, and the mean square error was 0.39 cm/a. In Table 4, seven points after GACOS correction had a reduced rate difference between GACOS correction and GPS, and six points, marked in red, showed an increased rate difference. The biggest error caused by GACOS was at GPS station SACY, and the error value was 0.23 cm/a.

In Figure 7, the points are consistent with the cumulative deformation of the GPS except for at point SBCC. Considering that the SBCC point unwrapping effect was poor, there may have been a gross error at this point, so it was removed, leaving 14 points in middle altitude for comparison. Among these, there were nine points after GACOS correction for which the difference was reduced, and four points showed increases. In Table 5, the biggest error caused by GACOS in the medium-elevation area was at GPS station CCCS, and the error value was 0.36 cm/a. The difference between SBAS-InSAR and GPS was from −0.45 cm/a to 0.67 cm/a, and the mean square error was 0.30cm/a. The difference between GACOS-based SBAS-InSAR and GPS was from −0.38 cm/a to 0.53 cm/a, and the mean square error was 0.23 cm/a. The accuracy was increased by about 23%.

In Figure 8, the time-series deformation of 15 points in the high-altitude region is shown. In Table 6, the difference between SBAS-InSAR and GPS was −0.49 cm/a to 0.38 cm/a, and the mean square error was 0.27 cm/a. The difference between GACOS-based SBAS-InSAR and GPS was −0.48 cm/a to 0.38 cm/a, and the mean square error was 0.26 cm/a. Among these points, there were eight for which the difference after GACOS correction was reduced, and there were six points with increased difference. The biggest error caused by GACOS in the high-elevation area was at RTHS, and the error value was 0.48 cm/a.

The relative errors in GACOS-based SBAS-InSAR and SBAS-InSAR with GPS in the LOS direction are compared in Table 7. Generally speaking, 24 points after GACOS correction showed decreased difference from GPS, and 16 points showed increased difference. The mean square error of SBAS-InSAR was 0.34 cm/a, and that of GACOS-based SBAS-InSAR was 0.31 cm/a, which shows that the GACOS correction improved the overall deformation monitoring accuracy but that the effect of improvement was limited. The improvement of the mid-altitude points was the most obvious after GACOS correction, while the accuracy for low- and high-altitude areas was roughly equal and there was no significant improvement. Additionally, GACOS correction tended to increase the error for all altitude points, and the maximum error caused by GACOS correction was 0.48 cm/a for high-altitude point RTHS. 

As we can see, there was no obvious correlation between the correction effect of GACOS products and the elevation; that is, the elevation-related component had no obvious influence. As mentioned above, a short-scale component introduced by turbulence is the other main component in the ITD model. The correction effect is likely to be related to the turbulence component in coastal and mountainous areas. As mentioned above, the ECMWF weather model for turbulent components has a spatiotemporal resolution of 0.125° and 6 h. The low resolution of the turbulence component presumably affected the result.

## 5. Conclusions

In this paper, GACOS products were assessed in SBAS-InSAR deformation monitoring, and, in theory, GACOS products can also be used in PS-InSAR. A total of 96 differential interferograms with vertical baselines of less than 200 m and time intervals of less than 300 days were used to generate time-series deformation maps of southern California from May 14, 2005 to September 25, 2010. Compared with classical SBAS-InSAR and GPS data, the GACOS-based SBAS-InSAR results showed that, in the whole range, after GACOS correction, the difference from GPS data decreased for 24 of the points examined and increased for 16 points. The mean square error decreased from 0.34 cm/a to 0.31 cm/a, and the accuracy of the time-series InSAR increased. In southern California, the accuracy of sequential InSAR was improved as a whole by GACOS atmospheric product correction, while GACOS correction increased the error for some points. In addition, GACOS atmospheric products had the best correction effect in the middle-elevation area far from the coastline with flat terrain, while the accuracy in the low-elevation and high-elevation areas was approximately the same, with no significant improvement. The correction effect may have been limited by the accuracy of turbulence data in GACOS products.

## Figures and Tables

**Table 1:** Basic parameters of the advanced synthetic aperture radar (ASAR) dataset.

Number	Satellite	Track Number	Imaging Time	$B_{⊥}\;(m)$
1	ENVISAT	16,757	20050514	0
2	ENVISAT	17,258	20050618	–198.77
3	ENVISAT	17,759	20050723	101.75
4	ENVISAT	18,260	20050827	124.26
5	ENVISAT	19,763	20051210	–441.12
6	ENVISAT	21,266	20060325	–242.02
7	ENVISAT	21,767	20060429	–197.76
8	ENVISAT	24,773	20061125	–603.90
9	ENVISAT	25,274	20061230	–98.02
10	ENVISAT	26,276	20070310	–47.40
11	ENVISAT	26,777	20070414	–641.67
12	ENVISAT	27,779	20070623	–455.47
13	ENVISAT	29,282	20071006	–624.64
14	ENVISAT	29,783	20071110	–279.92
15	ENVISAT	31,286	20080223	–724.89
16	ENVISAT	31,787	20080329	–194.16
17	ENVISAT	32,288	20080503	–539.79
18	ENVISAT	33,791	20080816	–349.14
19	ENVISAT	37,298	20090418	–629.36
20	ENVISAT	37,799	20090523	–427.74
21	ENVISAT	38,300	20090627	–224.70
22	ENVISAT	38,801	20090801	–532.88
23	ENVISAT	40,304	20091114	–274.60
24	ENVISAT	41,807	20100227	–717.09
25	ENVISAT	42,308	20100403	–89.48
26	ENVISAT	44,312	20100821	–587.65
27	ENVISAT	44,813	20100925	–244.90

**Table 2:** The positions of the global positioning system (GPS) points.

Name	Lat ^1^ (°)	Lon ^2^ (°)	Elev ^3^ (m)	Name	Lat (°)	Lon (°)	Elev (m)
AZU1	34.126	117.896	144.75	MAT2	33.857	117.437	398.30
BGIS	33.967	118.160	2.82	MHMS	33.939	118.244	−2.44
BKMS	33.962	118.095	11.00	MLFP	33.918	117.318	472.95
BLSA	33.800	118.029	−23.11	MTA1	34.055	118.246	72.65
CCCO	33.876	118.211	−16.93	OXYC	34.129	118.207	209.82
CCCS	33.863	117.865	31.82	PMHS	33.903	118.154	−11.13
CIT1	34.137	118.127	215.33	PVHS	33.779	118.372	259.58
CLAR	34.110	117.709	373.62	RTHS	34.089	117.353	328.67
CNPP	33.858	117.609	300.29	SACY	33.743	117.896	−11.24
CRHS	33.824	118.273	−23.55	SBCC	33.553	117.661	88.68
CSDH	33.861	118.257	−9.19	SGHS	34.089	118.109	79.86
CVHS	34.082	117.902	119.09	SILK	34.103	118.264	106.22
ELSC	34.030	118.208	61.19	SNHS	33.927	117.929	66.41
FVPK	33.662	117.936	−11.54	SPMS	33.993	117.849	207.03
GVRS	34.047	118.113	154.52	TORP	33.798	118.331	−5.22
HOLP	33.925	118.168	−6.67	TWMS	33.972	117.726	208.07
JPLM	34.205	118.173	424.00	USC1	34.024	118.285	21.93
LASC	33.928	118.307	24.67	VDCY	34.179	118.220	318.18
LBC1	33.832	118.137	−21.93	VTIS	33.713	118.294	59.49
LBC2	33.792	118.173	−28.49	VYAS	34.031	117.992	56.45
LBCH	33.788	118.203	−27.56	WCHS	34.062	117.911	100.10
LONG	34.112	118.003	74.27	WHC1	33.980	118.031	94.30
LORS	34.133	117.754	448.88				

^1^ “Lat” is the abbreviation of the word “Latitude”. ^2^ “Lon” is the abbreviation of the word “Longitude”. ^3^ “Elev” is the abbreviation of the word “Elevation”.

**Table 3:** GPS classification table.

Elevation	≤15 m	≤140 m and ≥15 m	≥140 m
Class	low	medium	high

**Table 4:** Mean rate comparison at low-altitude points (unit: cm/a).

Name	GPS	SBAS	GACOS-SBAS	GPS-SBAS	GPS-GACOS-SBAS
LBC2	−1.49	−1.08	−1.11	−0.41	−0.38
LBCH	−1.44	−1.00	−1.08	−0.44	−0.36
CRHS	−1.51	−1.04	−1.18	−0.47	−0.33
BLSA	−2.59	−2.10	−1.98	−0.49	−0.61
LBC1	−2.51	−1.98	−1.90	−0.53	−0.61
CCCO	−1.94	−1.55	−1.55	−0.39	−0.39
FVPK	−1.77	−1.30	−1.18	−0.47	−0.59
SACY	−2.24	−1.95	−1.72	−0.29	−0.52
PMHS	−1.43	−1.84	−1.72	0.41	0.29
CSDH	−1.41	−0.90	−0.98	−0.51	−0.43
HOLP	−1.92	−1.93	−1.82	0.01	−0.10
TORP	−1.59	−1.08	−1.34	−0.51	−0.25
MHMS	−1.87	−1.62	−1.62	−0.25	−0.25
BGIS	−1.77	−1.99	−1.82	0.22	0.05
BKMS	−1.70	−1.78	−1.52	0.08	−0.18

**Table 5:** Mean rate comparison at medium-elevation points.

Name	GPS	SBAS	GACOS-SBAS	GPS-SBAS	GPS-GACOS-SBAS
USC1	−1.66	−1.69	−1.69	0.03	0.03
LASC	−1.46	−1.45	−1.56	−0.01	0.10
CCCS	−1.70	−1.68	−1.32	−0.02	−0.38
VYAS	−1.49	−1.66	−1.29	0.17	−0.20
VTIS	−1.66	−1.21	−1.55	−0.45	−0.11
ELSC	−1.39	−1.75	−1.66	0.36	0.27
SNHS	−1.29	−1.54	−1.18	0.25	−0.11
MTA1	−1.60	−1.72	−1.70	0.12	0.10
LONG	−1.43	−2.10	−1.96	0.67	0.53
SGHS	−1.39	−1.80	−1.58	0.41	0.19
WHC1	−1.66	−1.84	−1.54	0.18	−0.12
WCHS	−1.64	−1.94	−1.54	0.30	−0.10
SILK	−1.43	−1.61	−1.63	0.18	0.20
CVHS	−1.75	−1.96	−1.56	0.21	−0.19

**Table 6:** Mean rate comparison at high-elevation points.

Name	GPS	SBAS	GACOS-SBAS	GPS-SBAS	GPS-GACOS-SBAS
AZU1	−1.44	−1.84	−1.47	0.40	0.03
GVRS	−1.58	−1.83	−1.66	0.25	0.08
SPMS	−1.44	−1.63	−1.30	0.19	−0.14
TWMS	−1.44	−1.34	−1.02	−0.10	−0.42
OXYC	−1.40	−1.78	−1.78	0.38	0.38
CIT1	−1.21	−1.59	−1.49	0.38	0.28
PVHS	−1.57	−1.08	−1.64	−0.49	0.07
CNPP	−1.32	−1.12	−0.93	−0.20	−0.39
VDCY	−1.64	−1.59	−1.71	−0.05	0.07
RTHS	−1.22	−1.22	−0.74	0.00	−0.48
CLAR	−0.96	−1.30	−1.02	0.34	0.06
MAT2	−1.22	−1.26	−1.06	0.04	−0.16
JPLM	−1.44	−1.63	−1.75	0.19	0.31
LORS	−1.34	−1.59	−1.35	0.25	0.01
MLFP	−1.24	−1.27	−0.92	0.03	−0.32

**Table 7:** Comparison of the two methods in the three elevation classes.

	Low	Medium	High	Total
Number of increased errors	6	4	6	16
Number of reduced errors	7	9	8	24
Maximum error (cm/a)	0.23	0.36	0.48	0.48
σ(SBAS-InSAR)	0.40	0.30	0.27	0.34
σ(GACOS-based SBAS-InSAR)	0.39	0.23	0.26	0.31
